# Patterns of Regional Brain Atrophy and Brain Aging in Middle- and Older-Aged Adults With Type 1 Diabetes

**DOI:** 10.1001/jamanetworkopen.2023.16182

**Published:** 2023-06-01

**Authors:** Mohamad Habes, Alan M. Jacobson, Barbara H. Braffett, Tanweer Rashid, Christopher M. Ryan, Haochang Shou, Yuhan Cui, Christos Davatzikos, Jose A. Luchsinger, Geert J. Biessels, Ionut Bebu, Rose A. Gubitosi-Klug, R. Nick Bryan, Ilya M. Nasrallah, and the DCCT/EDIC Research Group

**Affiliations:** 1 Neuroimage Analytics Laboratory (NAL) and the Biggs Institute Neuroimaging Core (BINC), Glenn Biggs Institute for Alzheimer's and Neurodegenerative Diseases, University of Texas Health Science Center San Antonio, San Antonio; 2 Center for Biomedical Image Computing and Analytics, Perelman School of Medicine, University of Pennsylvania, Philadelphia; 3 NYU Long Island School of Medicine, NYU Langone Hospital-Long Island, Mineola, New York; 4 George Washington University, Biostatistics Center, Rockville, Maryland; 5 University of Pittsburgh, Pittsburgh, Pennsylvania; 6 Department of Biostatistics, Epidemiology, and Informatics, University of Pennsylvania, Philadelphia; 7 Columbia University Irving Medical Center, New York, New York; 8 Department of Neurology, UMCU Brain Center, University Medical Center Utrecht, Utrecht, the Netherlands; 9 Case Western Reserve University School of Medicine, Rainbow Babies and Children's Hospital, Cleveland, Ohio

## Abstract

**Importance:**

Little is known about structural brain changes in type 1 diabetes (T1D) and whether there are early manifestations of a neurodegenerative condition like Alzheimer disease (AD) or evidence of premature brain aging.

**Objective:**

To evaluate neuroimaging markers of brain age and AD-like atrophy in participants with T1D in the Diabetes Control and Complications Trial (DCCT)/Epidemiology of Diabetes Interventions and Complications (EDIC) study, identify which brain regions are associated with the greatest changes in patients with T1D, and assess the association between cognition and brain aging indices.

**Design, Setting, and Participants:**

This cohort study leveraged data collected during the combined DCCT (randomized clinical trial, 1983-1993) and EDIC (observational study, 1994 to present) studies at 27 clinical centers in the US and Canada. A total of 416 eligible EDIC participants and 99 demographically similar adults without diabetes were enrolled in the magnetic resonance imaging (MRI) ancillary study, which reports cross-sectional data collected in 2018 to 2019 and relates it to factors measured longitudinally in DCCT/EDIC. Data analyses were performed between July 2020 and April 2022.

**Exposure:**

T1D diagnosis.

**Main Outcomes and Measures:**

Psychomotor and mental efficiency were evaluated using verbal fluency, digit symbol substitution test, trail making part B, and the grooved pegboard. Immediate memory scores were derived from the logical memory subtest of the Wechsler memory scale and the Wechsler digit symbol substitution test. MRI and machine learning indices were calculated to predict brain age and quantify AD-like atrophy.

**Results:**

This study included 416 EDIC participants with a median (range) age of 60 (44-74) years (87 of 416 [21%] were older than 65 years) and a median (range) diabetes duration of 37 (30-51) years. EDIC participants had consistently higher brain age values compared with controls without diabetes, indicative of approximately 6 additional years of brain aging (EDIC participants: β, 6.16; SE, 0.71; control participants: β, 1.04; SE, 0.04; *P* < .001). In contrast, AD regional atrophy was comparable between the 2 groups. Regions with atrophy in EDIC participants vs controls were observed mainly in the bilateral thalamus and putamen. Greater brain age was associated with lower psychomotor and mental efficiency among EDIC participants (β, −0.04; SE, 0.01; *P* < .001), but not among controls.

**Conclusions and Relevance:**

The findings of this study suggest an increase in brain aging among individuals with T1D without any early signs of AD-related neurodegeneration. These increases were associated with reduced cognitive performance, but overall, the abnormal patterns seen in this sample were modest, even after a mean of 38 years with T1D.

## Key Points

**Question:**

Is there radiographic evidence of premature brain aging in individuals with type 1 diabetes (T1D)?

**Findings:**

In this cohort study of 416 adults with T1D and 99 controls, participants with T1D had higher brain age values compared with controls, while Alzheimer disease–like regional atrophy was comparable between the 2 groups. Greater neuroimaging signs of brain aging were associated with lower psychomotor and mental efficiency among participants with T1D.

**Meaning:**

These findings suggest that individuals with T1D have advanced brain aging without any early signs of Alzheimer disease–related neurodegeneration compared with those without T1D.

## Teaser

This cohort study evaluates neuroimaging markers of brain age and Alzheimer disease–like atrophy in participants with type 1 diabetes and assesses the association between cognition and brain aging indices.

## Introduction

Modest structural and functional changes to the brain occur in children and young adults with type 1 diabetes (T1D).^1^ By the age of 60, some of these individuals show declines in performance on memory and mental efficiency tests^2^ and smaller gray matter volumes,^3^ potentially early signs of diabetes-associated dementia or mild cognitive impairment (MCI). It remains unclear which brain regions are most affected in T1D^4,5,6,7^ and whether these structural changes are an early manifestation of a neurodegenerative condition like Alzheimer disease (AD) or reflect an accelerated brain aging process.

We have previously developed machine learning–based strategies to differentiate brain aging from neurodegenerative processes by deriving indices from 10 216 harmonized brain MRI scans assembled for the Imaging-Based Coordinate System for Aging and Neurodegenerative Diseases (iSTAGING) consortium.^8^ These methods and data helped us identify a brain aging signature—a typical age-related gray matter atrophy pattern from cognitively normal adults across the adulthood lifespan,^9,10^ Spatial Pattern for Recognition–Brain Age (SPARE-BA) and Spatial Pattern for Recognition–Alzheimer disease (SPARE-AD), an AD-like atrophy pattern derived from amyloid-positive older adults with AD that can predict progression from normal cognition to MCI.^8,11,12,13,14^ Using these MRI-derived signatures, we can determine whether the brain structure of middle-aged and older-aged adults with a long history of T1D is similar to the pattern of aging vs early AD-like atrophy. The Diabetes Control and Complications Trial (DCCT)/Epidemiology of Diabetes Interventions and Complications (EDIC) study provides a unique opportunity to address 4 major aims: (1) to evaluate whether middle- and older-aged adults with T1D have advanced brain aging and greater AD-like atrophy compared with demographically similar adults without diabetes; (2) to identify which brain regions associated with the greatest changes in patients with T1D; (3) to examine the association between these atrophy patterns and diabetes-associated biomedical and metabolic characteristics in participants with T1D; and (4) to assess the association between cognition and brain atrophy patterns.

## Methods

For this cohort study, institutional review boards at all participating centers approved the protocol, and participants provided written informed consent. This study followed the Strengthening the Reporting of Observational Studies in Epidemiology (STROBE) reporting guideline.

### EDIC Participants

The DCCT randomized 1441 participants with T1D (between 1983 and 1989; mean [range] age, 27 [13-39] years) to receive intensive or conventional diabetes therapy with the goal of assessing treatment effects directed at achieving near-normal glycemia on the development and progression of diabetes-related complications.^15^ Baseline exclusion criteria included hypertension, hyperlipidemia, cardiovascular disease, neuropathy requiring medical intervention, and a history of recurrent severe hypoglycemia. The DCCT ended after a mean of 6.5 years of follow-up, having demonstrated the benefit of intensive glycemic therapy.^15^ In 1994, 96% of the surviving DCCT cohort enrolled in EDIC, an ongoing, long-term observational study.^16^ From 2018 to 2019, 425 of the 1190 actively participating EDIC participants without known end-stage renal disease, visual acuity worse than 20/40 corrected in both eyes, or a pacemaker implanted neurostimulator were randomly selected and invited to enroll in the EDIC MRI ancillary study. Additional exclusions included severe claustrophobia, known or suspected foreign metallic object in the body, or bodyweight greater than 350 lbs. The EDIC MRI study was conducted after a mean participant follow-up of 32 years.^3^

### Controls Without Diabetes

A demographically similar comparison group of adults without diabetes or serious current illnesses, including no prior history of stroke, was recruited from the community at each participating EDIC site. One hundred controls were matched to 100 randomly selected EDIC participants by race and ethnicity, age within 5 years older or younger, and educational attainment.^3^ Three controls with HbA1c levels of 6.5% or more and 1 with significant structural legions were excluded. Additionally, 2 EDIC participants with missing MRI data and 7 with significant structural legions were excluded. Missing data for cardiometabolic risk factors were less than 5%. The final sample included 416 EDIC participants and 99 controls.

### Evaluations, Risk Factors, and Coexisting Complications

Participants were asked to self-report their predominant race and ethnicity during an interview-administered survey at DCCT baseline. The form that was used was created in 1982 and 1983 and included the following categories—American Indian or Alaskan Native, Asian or Pacific Islander, Hispanic, non-Hispanic Black, and non-Hispanic White. Race and ethnicity data were collected to describe the cohort. Diabetes-related and cardiovascular risk factors were assessed in EDIC participants and controls without diabetes by standardized methods.^15,16^ Measurements of risk factors were performed longitudinally for EDIC participants (quarterly during DCCT, annually during EDIC) and cross-sectionally for controls at the time of the MRI study. A detailed medical history was obtained, including demographic factors, medications, and medical outcomes. A physical examination measured height, weight, body mass index (BMI; calculated as weight in kilograms divided by height in meters squared), sitting blood pressure, waist circumference, and pulse rate.^15,16^ Laboratory studies included fasting lipids, albumin excretion rate (AER), HbA1c by high-performance liquid chromatography, and, for EDIC participants, serum creatinine. Hypertension was defined as systolic blood pressure of 140 mm Hg or higher, diastolic blood pressure of 90 mm Hg or higher, documented hypertension, or antihypertensive medication use. Hyperlipidemia was defined as low density lipoprotein cholesterol of 130 mg/dL (to convert to millimoles per liter, multiply by 0.0259) or higher or lipid-lowering medication use. Measures of diabetes-related complications ascertained in EDIC have been previously described^17,18,19,20^ (eMethods in Supplement 1).

### Cognitive Protocol

Cognitive assessments were conducted longitudinally during the DCCT/EDIC study and have been described previously.^2,21,22^ The most recent assessment was performed at the time of the MRI study, after a mean of 32 years of follow-up, and included an abbreviated battery consisting of a subset of psychomotor and mental efficiency tests found to be particularly sensitive to diabetes,^22,23^ and tests of memory known to be sensitive to normal aging and mild cognitive impairment.^24^ Psychomotor and mental efficiency were evaluated using verbal fluency, digit symbol substitution test, trail making part B, and the grooved pegboard. Immediate memory scores were derived from the logical memory subtest of the Wechsler memory scale and the Wechsler digit symbol substitution test. The delayed recall was assessed by the recall of logical memory stories after a 10- to 15-minute delay. Cognitive tests were acquired within a mean of 46 days after the MRI, with 66% occurring within 7 days. For both EDIC participants and controls, a standardized *z* score was calculated for each of the test variables using the mean and SDs of the DCCT/EDIC cohort from the DCCT baseline evaluation. A summary score was obtained by taking the average of all *z* scores in each domain. These standardized scores provide a unit-free measurement of the relative difference in performance as compared with the total DCCT/EDIC cohort at the referent DCCT baseline assessment. For each domain, the simple mean of the standardized scores represents the change from baseline, with equal weight assigned to each test within the domain.^2,22^

### Imaging Protocols and Image Preprocessing

The MRI neuroimaging component has been described previously.^3^ Briefly, MRI scanning was performed at 24 imaging centers (26 of 27 EDIC sites) using Siemens, Philips, and GE 3 Tesla scanners. Imaging parameters for Siemens and GE scanners included a field of view of 250 mm, 176 slices, and a native resolution of 1 mm isotropic. The imaging parameters for the Philips scanner included a field of view of 256 mm, 170 slices, and a native resolution of 1 mm isotropic. Scanner performance was monitored with quarterly Alzheimer Disease Neuroimaging Initiative (ADNI) phantom analyses, with all scanners showing stability of measurements during the study. Eight scans (7 EDIC participants, 1 control) were excluded from analyses due to significant structural lesions that affected outcome measures: 5 encephalomalacia, 1 meningioma with mass effect, 1 neurodevelopmental abnormality, and 1 likely multiple sclerosis.

T1-weighted images were first corrected for intensity bias.^25^ Next, a multi-atlas segmentation method^26^ was applied to strip skulls and extract the brain from surrounding tissues. The skull-stripped T1 brain images were then segmented into a number of anatomical regions of interest (ROIs) by a robust multi-atlas label fusion method.^27^

### Harmonization of Regions of Interest Volumes

We applied our previously developed statistical harmonization pipeline to remove scanner-related differences by adjusting location (mean) and scale (variance) effects.^28^ We harmonized each ROI across EDIC sites using age, sex, intracranial volume (ICV), and diagnosis as covariates using Combat-GAM (generalized additive model) harmonization to remove scanner variation.^30^ Since MRI data in EDIC were collected using multiple scanners, we first performed Combat-GAM harmonization to remove scanner variation between the multiple scanners used in the EDIC MRI study. To ensure machine learning model generalization and consistency, we employed a second Combat-GAM harmonization on the EDIC participant data against control data from other studies assembled as part of a separate and larger consortium on trajectories of neuroimaging biomarkers in aging and neurodegeneration (iSTAGING). A second harmonization permitted the application of robust SPARE-BA and -AD models developed in the iSTAGING space.^8^ From iSTAGING, we included 2764 cognitively healthy individuals with no clinical diagnosis of diabetes from the following studies: 37 participants from the Australian Imaging, Biomarker, and Lifestyle Flagship Study of Aging study, 649 participants from the Coronary Artery Risk Development in Young Adults study, and 2078 participants from the UK-Biobank study. The harmonization model included age, sex, and diagnosis as covariates and allowed identification of nonlinear trends in ROI volumes.

### SPARE Indices

We derived SPARE indices from the 2-step harmonized ROI data to measure predicted brain age (SPARE-BA) using a machine learning method based on Support Vector Regression.^8,11,13^ The SPARE-BA model was previously trained on the large iSTAGING control sample. Higher SPARE-BA relative to chronologic age indicates more age-related atrophy. We measured atrophy in regions affected in AD using SPARE-AD,^8,11,13^ which was derived using a support vector machine with linear kernel and trained to identify differences between controls without AD using data from the ADNI study. The SPARE-AD model on 256 harmonized iSTAGING control participants was built with negative cerebral amyloid status and 221 AD participants with positive cerebral amyloid status. Positive and higher SPARE-AD values point to more AD-like atrophy, while negative and lower values indicate normal brain patterns. More details on the SPARE indices are provided in the eMethods in Supplement 1.

### Statistical Analysis

Differences in demographic and clinical characteristics between EDIC participants and controls were tested using the Wilcoxon rank-sum test for quantitative characteristics or the χ^2^ test for categorical characteristics. Linear mixed models were used to estimate mean differences in SPARE-BA and SPARE-AD between groups. Among EDIC participants only, we used linear regression models to assess covariate effects on the mean of each MRI outcome. Quantitative covariates were characterized by the time-weighted mean of all DCCT/EDIC follow-up values from the DCCT baseline to the MRI visit, weighting each value by the time interval since the last measurement. Categorical covariates, other than sex, were defined as any report prior to the MRI visit. Comprehensive multivariable regression models were developed for each outcome using a backward elimination, where variables significant at *P* < .10 were retained at each step. The final multivariable models retained covariates significant at *P* < .05. Signed *t *values are presented and correspond to the magnitude and directionality of the association. With our large sample size, *t *values and z-values converge to a normal distribution. Both are used to differentiate covariate effects with a *P* < .001 (2-sided) equivalent to a |Z| of 3.89 or more. All models were adjusted for age, sex, and scanner. We estimated the additional number of years of age that would yield the same difference in each MRI outcome as the difference between EDIC and control participants. We found this by taking the ratio of the β coefficient estimate for the participant group to that of age from a linear mixed model that included both factors, with adjustment for ICV and scanner.

Separately for EDIC participants and controls, linear regression models were used to evaluate the individual associations of each MRI measure (independent variable) with a summary z-score for each cognitive domain, adjusting for age, sex, years of education, and scanner. Finally, similar linear regression models were used to evaluate differences in brain ROIs between groups as well as to assess associations between risk factors and ROIs among only EDIC participants. Results with false discovery rate (FDR) values less than 0.05 were considered significant. All analyses were performed using SAS software version 9.4 (SAS Institute). Data analyses were performed between July 2020 and April 2022.

## Results

### Participants

This study included 416 EDIC participants with a median (range) age of 60 (44-74) years (87 of 416 [21%] were older than 65 years) and a median (range) diabetes duration of 37 (30-51) years. The 99 control participants included had a significantly greater attained education (16.2 [1.5] years vs 15.6 [1.9] years; *P* = .02) but otherwise were similar to the EDIC participants with no significant differences in other demographic variables (eTable 1 in Supplement 1). EDIC participants had significantly lower diastolic blood pressure values and more favorable lipid profiles, possibly related to the exclusion of individuals with hypertension and dyslipidemia at DCCT baseline and the subsequent assiduous care by their health care providers to mitigate risk for cardiovascular disease.

### SPARE Indices of Brain Atrophy

**Figure 1.**
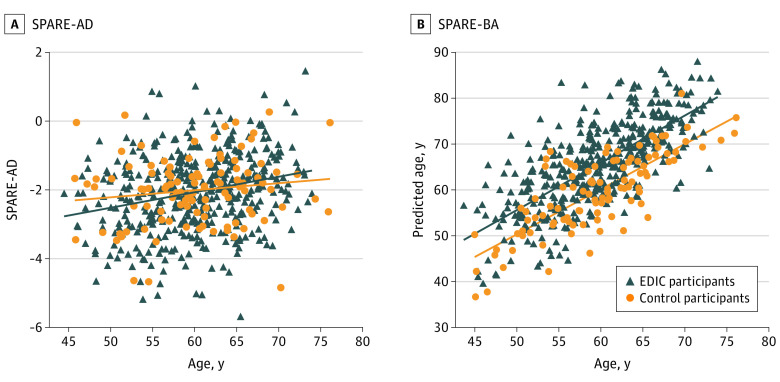
Machine Learning Indices, SPARE-AD and SPARE-BA, as a Function of Age A, there was no significant difference observed between EDIC participants (blue) and controls without diabetes (orange). In panel B, EDIC participants showed a significant increase in predicted brain age (SPARE-BA) demonstrating more advanced brain aging patterns. SPARE-AD indicates spatial pattern for recognition-Alzheimer disease; SPARE-BA, spatial pattern for recognition-brain age.

Figure 1 illustrates SPARE-BA and SPARE-AD scores for EDIC participants and controls without diabetes. Across the entire actual age range, EDIC participants had consistently higher predicted age (SPARE-BA) values compared with controls, indicative of approximately 6 additional years of brain aging (EDIC participants: β, 6.16; SE, 0.71; control participants: β, 1.04; SE, 0.04; *P* < .001) (Figure 1). In contrast, SPARE-AD values were comparable between the 2 groups, suggesting that, at least within this middle age and older age range, there is no greater atrophy in regions typically affected in AD (Figure 1).

**Figure 2.**
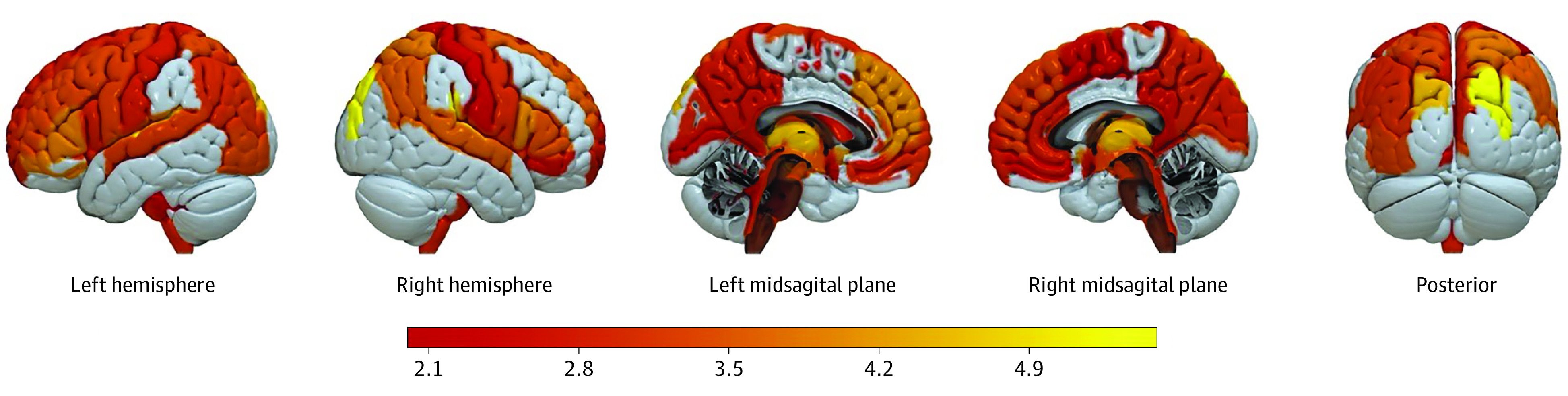
Patterns of Reduced Gray Matter in Patients With Type 1 Diabetes Epidemiology of Diabetes Interventions and Complications study participants displayed widespread differences in atrophy patterns, most pronounced in the superior frontal gyrus, middle frontal gyrus and superior temporal gyrus, as well as the putamen, thalamus.

To identify which brain regions have been altered due to T1D, we calculated differences in effect sizes between EDIC participants and controls across ROIs. Figure 2 shows the regions with significant atrophy in EDIC participants vs controls, with the most atrophy observed in the bilateral planum temporale, bilateral superior occipital gyrus, right transverse temporal gyrus, and bilateral thalamus, putamen, and pallidum. Most temporal lobe ROIs, which have particularly important influences on SPARE-AD, did not show significant between-group differences. eTable 2 in Supplement 1 lists all the ROIs and their corresponding FDR-corrected *P* values and effect sizes.

### Associations With Risk Factors

Among EDIC participants, SPARE-BA and SPARE-AD were not associated with measures of glycemia or with measures of diabetes-related complications, such as neuropathy, retinopathy, and kidney disease (Table 1). Hypertension and hyperlipidemia were common in EDIC participants, but were well-controlled, and neither were associated with the SPARE measures. Increased BMI (SPARE-AD: β, −0.04; SE, 0.01; *P* = .01; SPARE-BA: β, −0.23; SE, 0.09; *P* = .007) and waist circumference (SPARE-AD: β, −0.06; SE, 0.02; *P* = .005; SPARE-BA: β, −0.27; SE, 0.12; *P* = .03) were associated with less AD-like atrophy and brain age-related atrophy. BMI remained a significant factor associated with SPARE-BA (β, −0.04; SE, 0.01; *P* = .01) and SPARE-AD (β, −0.32; SE, 0.09; *P* < .001) in multivariable models (Table 2). Multivariable models showed that higher diastolic blood pressure was associated with SPARE-BA (β, 0.18; SE, 0.07; *P* = .01) but not with SPARE-AD in EDIC participants (eTable 3 in Supplement 1). We found few significant associations between the ROIs most significantly affected by T1D (those shown in Figure 2 but with effect sizes >3) and HbA1c, systolic blood pressure, or cumulative severe hypoglycemia events (eTable 4 in Supplement 1).

**Table 1.** Association of Traditional Glycemic and Nonglycemic Risk Factors and Microvascular and Macrovascular Complications With MRI Outcomes Among 416 EDIC Participants, Adjusted for Age, Sex, and Scanner

Characteristic	SPARE-AD^a^			SPARE-BA^a^		
	β (SE)	*t*	*P* value	β (SE)	*t*	*P* value
Demographic						
Education, per 1 y	0.01 (0.03)	0.34	.74	−0.25 (0.17)	−1.5	.13
Sex, male vs female	−0.36 (0.11)	−3.36	.001	−0.62 (0.63)	−0.98	.33
Risk factors						
Glycemic						
Hemoglobin A1c, per 1 %^b^	−0.01 (0.06)	−0.17	.86	0.31 (0.38)	0.83	.41
Severe hypoglycemia						
Cumulative, ≥1 vs 0 events^c^	−0.06 (0.11)	−0.57	.57	1.07 (0.63)	1.69	.09
1-5 vs 0 events	−0.09 (0.12)	−0.81	.42	0.93 (0.68)	1.37	.17
>5 vs 0 events	0.06 (0.19)	0.33	.74	1.59 (1.14)	1.4	.16
Nonglycemic						
BMI^b^	−0.04 (0.01)	−2.49	.01	−0.23 (0.09)	−2.74	.007
Waist circumference, per 5 cm	−0.06 (0.02)	−2.8	.005	−0.27 (0.12)	−2.19	.03
Blood pressure, per 5 mm Hg^b^						
Systolic	−0.01 (0.04)	−0.41	.68	0.34 (0.21)	1.64	.10
Diastolic	−0.02 (0.06)	−0.43	.67	0.5 (0.34)	1.5	.13
Any treated hypertension, yes vs no	−0.15 (0.16)	−0.95	.34	1.31 (0.94)	1.4	.16
Pulse rate, per 1 bpm^b^	0 (0.01)	−0.42	.67	0.03 (0.05)	0.66	.52
Plasma lipids^a^						
HDL/LDL ratio, per 0.1	0.04 (0.03)	1.47	.14	0.32 (0.17)	1.95	.05
Triglycerides, log	−0.18 (0.14)	−1.28	.20	−0.57 (0.85)	−0.67	.50
Any treated hyperlipidemia, yes vs no	0.13 (0.16)	0.85	.40	0.53 (0.93)	0.58	.56
Complications						
Kidney disease						
Sustained AER ≥ 30 mg/24 hr, yes vs no^d^	−0.05 (0.13)	−0.43	.67	0.84 (0.75)	1.11	.27
eGFR < 60 mL/min/1.73 m^2^, yes vs no^d^	0.01 (0.19)	0.03	.98	−0.37 (1.11)	−0.33	.74
Retinopathy						
PDR (yes vs no)^d^	−0.11 (0.13)	−0.87	.39	0.11 (0.74)	0.15	.88
CSME (yes vs no)^d^	−0.19 (0.12)	−1.56	.12	0.46 (0.71)	0.65	.52
Neuropathy						
Confirmed clinical neuropathy (yes vs no)^d^	−0.13 (0.12)	−1.1	.27	0.9 (0.71)	1.26	.21
Cardiovascular autonomic neuropathy (yes vs no)^d^	0.14 (0.11)	1.26	.21	1.29 (0.65)	1.97	.05
Cardiovascular disease, yes vs no^d^	−0.08 (0.16)	−0.49	.62	0.9 (0.94)	0.96	.34

Abbreviations: AER, albumin excretion rate; BMI, body mass index (calculated as weight in kilograms divided by height in meters squared); CSME, clinically significant macular edema; DCCT, Diabetes Control and Complications Trial; EDIC, Epidemiology of Diabetes Interventions and Complications; eGFR, estimated glomerular filtration; HDL, high-density lipoprotein; LDL, low-density lipoprotein; MRI, magnetic resonance imaging; PDR, proliferative diabetic retinopathy; SPARE-AD, spatial pattern for recognition-Alzheimer disease; SPARE-BA, spatial pattern for recognition-brain age.

^a^ Data are β coefficients, standard errors, *t *values, and *P* values from individual linear regression models evaluating the association of each covariate of interest (independent) with each MRI outcome (dependent), with adjustment for age, sex, and scanner. β estimates are equal to the difference in means between groups or the slope of the association (eg, increase or decrease in MRI outcome for every unit change in the covariate). The signed *t* value corresponds to the magnitude and directionality of the association.

^b^ Risk factors were characterized by the time-weighted mean values of all follow-up values since DCCT baseline up to the MRI study visit.

^c^ Severe hypoglycemia was defined as events leading to coma or seizure documented by self-report for the 3-month period prior to each visit.

^d^ Any report between DCCT baseline and the MRI study visit.

**Table 2.** Multivariable Models For MRI Outcomes Among EDIC Participants

Characteristic	EDIC participants, n = 416							
	SPARE-AD^a^				SPARE-BA^a^			
	β	SE	*t*	*P* value	B	SE	*t*	*P* value
Age (per 1 y)	0.04	0.01	5.15	<.001	1.03	0.05	21.03	<.001
Sex (men vs women)	−0.35	0.11	−3.27	.001	−1.19	0.68	−1.75	.08
BMI^b^	−0.04	0.01	−2.49	.01	−0.32	0.09	−3.49	.001
Diastolic blood pressure (per 5 mm Hg)^b^	NA	NA	NA	NA	0.18	0.07	2.59	.01

Abbreviations: BMI, body mass index (calculated as weight in kilograms divided by height in meters squared); DCCT, Diabetes Control and Complications Trial; EDIC, Epidemiology of Diabetes Interventions and Complications; MRI, magnetic resonance imaging; NA, not applicable; SPARE-AD, spatial pattern for recognition-Alzheimer disease; SPARE-BA, spatial pattern for recognition-brain age.

^a^ Data are β coefficients, standard errors, *t *values, and *P* values from 3 separate multivariable regression models evaluating the association of all the risk factors entered into the model together with each MRI outcome (dependent), and with further adjustment for age, sex, and scanner. Covariates that did not enter into any of the 3 models were not included in the table. β estimates are equal to the difference in means between groups or the slope of the association (eg, increase or decrease in MRI outcome for every unit change in the covariate). The signed *t *value corresponds to the magnitude and directionality of the association.

^b^ Risk factors were characterized by the time-weighted mean values of all follow-up values since DCCT baseline up to the MRI study visit.

### Associations With Cognitive Testing

Performance on cognitive measures correlated strongly with the 2 machine learning indices. Among EDIC participants, greater brain aging (SPARE-BA) was associated with lower psychomotor and mental efficiency (β, −0.04; SE, 0.01; *P* < .001) (Table 3), whereas greater SPARE-AD was associated with decreased psychomotor and mental efficiency (β, −0.17; SE, 0.04; *P* < .001), as well as with immediate (β, −0.13; SE, 0.04; *P* = .001) and delayed recall (β, −0.11; SE, 0.05; *P* = .02). The only significant association among controls was between SPARE-BA and delayed recall (β, −0.04; SE, 0.02; *P* = .03) (Table 3). Exploratory analyses of relationships of ROI volumes with cognitive scores demonstrated that these were driven by a limited number of ROIs rather than across wide brain regions. Psychomotor and mental efficiency scores were associated with volumes of the superior temporal gyrus, planum temporale, parietal operculum, thalamus proper area, as well as middle frontal gyrus and angular gyrus (eTable 5 in Supplement 1). Memory and delayed recall were not significantly associated with specific ROIs.

**Table 3.** Association of MRI Measures With Cognitive Domains Among EDIC Participants and Controls Without Diabetes, Adjusted For Age, Sex, Years of Education, and Scanner

Cohort	Immediate memory^a^			Delayed recall^a^			Psychomotor and mental efficiency^a^		
	β (SE)	*t*	*P* value	β (SE)	*t*	*P* value	β (SE)	*t*	*P* value
EDIC participants, n = 415									
SPARE-AD	−0.13 (0.04)	−3.26	.001	−0.11 (0.01)	−2.44	.02	−0.17 (0.05)	−3.66	<.001
SPARE-BA	−0.01 (0.01)	−0.72	.47	−0.01 (0.01)	−1.65	.1	−0.04 (0.01)	−4.96	<.001
Controls, n = 94									
SPARE-AD	−0.16 (0.11)	−1.4	.16	−0.133 (0.12)	−1.12	.27	−0.12 (0.10)	−1.26	.21
SPARE-BA	−0.03 (0.02)	−1.4	.16	−0.04 (0.02)	−2.16	.03	0.006 (0.02)	0.35	.73

Abbreviations: EDIC, Epidemiology of Diabetes Interventions and Complications; MRI, magnetic resonance imaging; SPARE-AD, spatial pattern for recognition-Alzheimer disease; SPARE-BA, spatial pattern for recognition-brain age.

^a^ Data are β coefficients, standard errors, t-values, and *P* values from individual linear regression models evaluating the association of each MRI measure (independent) with each cognitive domain (dependent), with adjustment for age, sex, years of education, and scanner. β estimates are equal to the slope of the association (eg, increase or decrease in cognitive domain for every unit change in the covariate). The signed *t *value corresponds to the magnitude and directionality of the association.

## Discussion

In this cohort study, we used novel machine learning methods to identify spatial patterns of brain atrophy and found that T1D was associated with an increase in brain age relative to individuals without diabetes. TID was not associated with a pattern consistent with early AD-related neurodegeneration. Our data suggest that, on average, individuals with T1D have brain atrophy patterns that were equivalent to approximately 6 years older age compared with the participants' chronological age, while controls without T1D showed no evidence of premature brain aging. These results support the hypothesis that brain morphology is associated with an accelerating aging process in middle-aged and older-aged adults with a long history of T1D.

Our study suggested that T1D was associated with pronounced gray matter atrophy in the putamen, thalamus, superior frontal gyrus, middle frontal gyrus, and superior temporal gyrus. These regions are known to provide important information for the SPARE-BA measure (Figure 2).^8^ The relatively parallel trendlines of SPARE-BA for T1D participants vs controls, suggest that this acceleration might have happened earlier in life than the age of 45 years old (Figure 1). The mechanism for premature brain aging in T1D requires additional investigation. Prior studies of brain atrophy in T1D have shown mixed findings. A meta-analysis of 10 studies with a combined sample size of 613 individuals showed evidence for thalamic atrophy in T1D.^29^ Our study confirmed this finding and identified additional regions affected in T1D, perhaps due to better harmonization of the imaging protocol and postprocessing harmonization. Prior studies have not found strong evidence that T1D is associated with hippocampal atrophy,^29^ which is consistent with our observation of hippocampal volume and the SPARE-AD result.

We did not assay for amyloid and tau biomarkers in the EDIC study to directly evaluate the prevalence of AD neuropathologic change. However, the EDIC participants with T1D and control participants without diabetes had comparable measures of atrophy in AD-signature regions, with both showing mean SPARE-AD values in the range of normal controls. This suggests that T1D is not associated with significantly decreased brain reserve in regions that are susceptible to AD-related neurodegeneration at this age. Risk factors, spanning demographic measures to vascular risk factors to diabetes-related complications, did not show significant associations with SPARE-AD or SPARE-BA measures, failing to identify a potential direct mechanism for the effects of T1D on brain health.

Previously in the iSTAGING sample, we found that advanced brain aging patterns in controls without T1D were associated with lower executive function but not worse memory performance. In contrast, higher SPARE-AD, characterized by a pattern showing greater atrophy in temporal lobe regions, was associated with both executive function and memory. In EDIC participants, SPARE-AD atrophy patterns seem to be associated with psychomotor and mental efficiency as well as memory. Brain aging was only associated with worse psychomotor and mental efficiency. These findings support the hypothesis that different regional atrophy patterns are associated with different cognitive impairment profiles.

### Limitations

This study had limitations. The major weakness of the study is the predominantly non-Hispanic White population which, while typical for type 1 diabetes in the US, limits the generalizability to other populations. Additionally, EDIC participants, who were volunteers initially enrolled in a clinical trial and subsequently in a long-term follow-up observation study, may not be representative of most individuals with T1D. However, they have been part of an observational study and been managed in the health care setting for the most of the follow-up period. Lastly, the cohort is at an age where the prevalence of AD pathology is expected to be low; this study does not address combinatorial effects of diabetes and AD pathology.

## Conclusions

The findings of this cohort study suggest that individuals with T1D show an acceleration of brain aging without any early signs of AD-related neurodegeneration. Regional atrophy is most pronounced in the thalamus. Brain atrophy is linked to changes in cognition, but overall, the differences seen in middle-aged to older-aged adults with T1D compared with controls without T1D were modest, even after more than a mean of 38 years of T1D.

## Notes

Supplement 1.
eMethods.
**eTable 1.** Characteristics of EDIC Participants and Controls Without Diabetes Enrolled in the MRI Study (2018-2019) **eTable 2.** Differences in Regions of Interest (ROI) Volumes Between EDIC Participants and Controls Without Diabetes **eTable 3. **Association of Traditional Glycemic and Nonglycemic Risk Factors and Microvascular and Macrovascular Complications With MRI Outcomes Among EDIC Participants, Unadjusted **eTable 4.** Association of HbA1c, SBP, and Hypoglycemia With Paired and Unpaired ROI’s **eTable 5.** Association of Paired ROI’s With Immediate Memory and Psychomotor and Mental Efficiency **eAppendix.** DCCT/EDIC Research Group

Supplement 2.
Data Sharing Statement
