# Supplementary material for: Patterns of Regional Brain Atrophy and Brain Aging in Middle- and Older-Aged Adults With Type 1 Diabetes
Source: JAMA Netw Open. 2023 Jun 1;6(6):e2316182. doi: 10.1001/jamanetworkopen.2023.16182 (PMC10236234; doi:10.1001/jamanetworkopen.2023.16182)
Supplement: Supplement 1. — eMethods. eTable 1. Characteristics of EDIC Participants and Controls Without Diabetes Enrolled in the MRI Study (2018-2019) eTable 2. Differences in Regions of Interest (ROI) Volumes Between EDIC Participants and Controls Without Diabetes eTable 3. Association of Traditional Glycemic and Nonglycemic Risk Factors and Microvascular and Macrovascular Complications With MRI Outcomes Among EDIC Participants, Unadjusted eTable 4. Association of HbA1c, SBP, and Hypoglycemia With Paired and Unpaired ROI’s eTable 5. Association of Paired ROI’s With Immediate Memory and Psychomotor and Mental Efficiency eAppendix. DCCT/EDIC Research Group [file jamanetwopen-e2316182-s001.pdf]

Supplemental Online Content

Habes M, Jacobson AM, Braffett BH, et al. Patterns of regional brain atrophy and brain aging in middle- and older-aged adults with type 1 diabetes. *JAMA Netw Open*. 2023;6(6):e2316182. doi:10.1001/jamanetworkopen.2023.16182

eMethods.

eTable 1. Characteristics of EDIC Participants and Controls Without Diabetes Enrolled in the MRI Study (2018-2019)

eTable 2. Differences in Regions of Interest (ROI) Volumes Between EDIC Participants and Controls Without Diabetes

eTable 3. Association of Traditional Glycemic and Nonglycemic Risk Factors and Microvascular and Macrovascular Complications With MRI Outcomes Among EDIC Participants, Unadjusted

eTable 4. Association of HbA1c, SBP, and Hypoglycemia With Paired and Unpaired ROI's

eTable 5. Association of Paired ROI's With Immediate Memory and Psychomotor and Mental Efficiency

eAppendix. DCCT/EDIC Research Group

This supplemental material has been provided by the authors to give readers additional information about their work.

© 2023 Habes M et al. *JAMA Network Open.*

eMethods.

Measures of diabetes-related complications

Among EDIC participants, severe hypoglycemia was defined as the cumulative number of events leading to coma or seizure within the 3-months prior to each study visit by self-report. Estimated glomerular filtration rates (eGFR) were calculated from serum creatinine and estimated with the Chronic Kidney Disease Epidemiology Collaboration equation. Kidney disease was defined as an AER ≥30 mg/24 hr on ≥two consecutive visits or eGFR <60 mL/min/1.73m^2, at any time during the DCCT/EDIC study[1]. Proliferative diabetic retinopathy was defined by neovascularization observed on standardized stereoscopic seven-field fundus photography grading or evidence of scatter photocoagulation. Clinically-significant macular edema was defined using fundus photography grading for evidence of macular thickening or the presence of focal photocoagulation scars, at any time during the DCCT/EDIC study[2]. Neurologic evaluations, nerve conduction studies, and cardiac autonomic testing were conducted periodically[3]. All cardiovascular disease (CVD) events were adjudicated and classified by a committee masked to DCCT treatment group assignment and HbA1c levels[4].

SPARE indices

We calculated an imaging estimate of the AD-like atrophy present on a brain MR scan named the SPARE-AD index, using models described in detail before[5]. The model has been previously described[5,6] and was constructed to maximally differentiate between clinical AD MR scans and cognitively healthy controls using a support vector machine. More positive SPARE-AD implies a more AD-like brain structure, while more negative values reflect more normal brain structure. For SPARE-AD calculation, we trained an SVM classifier with a linear kernel to predict the diagnosis status as either CN or AD. The training set included only CNs with known-negative Aβ status and only ADs with known-positive Aβ status. Studies were included in the training

© 2023 Habes M et al. *JAMA Network Open.*

set if they contained more than 30 subjects of each class after filtering for known Aβ status. The resulting training set is summarized as:

STUDY	N	CN	AD
ADNI-1	154	61	93
ADNI-2	323	195	128
TOTAL:	477	256	221

For predicted classes of the training set, we performed 10-fold cross-validation, using the predictions from each holdout fold to avoid over-fitting. The SPARE-AD classifier attains ROC Area-under-the-curve score of 0.948 and Accuracy of 0.893 based on the cross-validated predictions.

We calculated a brain-aging signature to estimate deviations from typical aging[7]. We developed a multivariate pattern regression model based on support vector regression to predict individualized brain age for each participant, which we defined as the Spatial Patterns of Brain Alteration for capturing Brain Aging (SPARE-BA) similar to our previous work[6,8]. The model was trained with the T1-MR scans using regional volumetric measures for structures. The brain age prediction regression made use of a radial basis function kernel. The gamma parameter was set to 0.1. Cost and epsilon parameters were kept at their defaults of 1.0 and 0.1, respectively. The training set included only cognitively normal subjects. For subjects in the training set, we performed stratified 10-fold cross-validation, stratifying on the study to preserve the relative proportion of studies in each fold. The SPARE-BA prediction regression was run after harmonization. The mean absolute error (MAE) of age predictions was 5.838.

© 2023 Habes M et al. *JAMA Network Open.*

REFERENCES

1. Perkins BA, Bebu I, de Boer IH, et al. Risk Factors for Kidney Disease in Type 1 Diabetes. *Diabetes Care*. 2019;42(5):883-890. doi:10.2337/dc18-2062

2. Hainsworth DP, Bebu I, Aiello LP, et al. Risk Factors for Retinopathy in Type 1 Diabetes: The DCCT/EDIC Study. *Diabetes Care*. 2019;42(5):875-882. doi:10.2337/dc18-2308

3. Albers JW, Herman WH, Pop-Busui R, et al. Effect of prior intensive insulin treatment during the Diabetes Control and Complications Trial (DCCT) on peripheral neuropathy in type 1 diabetes during the Epidemiology of Diabetes Interventions and Complications (EDIC) Study. *Diabetes Care*. 2010;33(5):1090-1096. doi:10.2337/dc09-1941

4. Nathan DM, Cleary PA, Backlund JYC, et al. Intensive diabetes treatment and cardiovascular disease in patients with type 1 diabetes. *N Engl J Med*. 2005;353(25):2643-2653. doi:10.1056/NEJMoa052187

5. Da X, Toledo JB, Zee J, et al. Integration and relative value of biomarkers for prediction of MCI to AD progression: Spatial patterns of brain atrophy, cognitive scores, APOE genotype and CSF biomarkers. *NeuroImage: Clinical*. 2014;4(0):164-173. doi:http://dx.doi.org/10.1016/j.nicl.2013.11.010

6. Habes M, Janowitz D, Erus G, et al. Advanced Brain Aging: relationship with epidemiologic and genetic risk factors, and overlap with Alzheimer disease atrophy patterns. *Transl Psychiatry*. 2016;6:e775.

7. Habes M, Pomponio R, Shou H, et al. The Brain Chart of Aging: Machine-learning analytics reveals links between brain aging, white matter disease, amyloid burden, and cognition in the iSTAGING consortium of 10,216 harmonized MR scans. *Alzheimers Dement*. 2021;17(1):89-102. doi:10.1002/alz.12178

8. Eavani H, Habes M, Satterthwaite TD, et al. Heterogeneity of structural and functional imaging patterns of advanced brain aging revealed via machine learning methods. *Neurobiol Aging*. 2018;71:41-50. doi:10.1016/j.neurobiolaging.2018.06.013

© 2023 Habes M et al. *JAMA Network Open.*

eTable 1. Characteristics of EDIC participants and controls without diabetes enrolled in the MRI study (2018-2019)

	EDIC Participants	Controls	p-value [a]
N	**416**	**99**	
**Demographic**			
Age at diagnosis of T1D (years)	21.8 ± 7.7	---	---
Age (years)	59.6 ± 6.4	59.8 ± 6.9	0.75
Age [median (range)]	60 (44-74)	60 (45-76)	
Age >65 years (%)	20.9	23.2	0.61
Female sex (%)	44.2	53.5	0.10
White race (%)	96.6	96.7	0.96
Education (years)	15.6 ± 1.9	16.2 ± 1.5	0.02
Professional or technical occupation (%)	58.7	66.0	0.19
Married or remarried (%)	81.9	84.5	0.54
**Behavioral**			
Current cigarette smoker (%)	8.0	5.1	0.32
Occasional or regular alcohol use (%)	53.5	64.7	0.05
**Physical**			
Body mass index (kg/m^2)	28.2 ± 5.1	27.9 ± 4.7	0.69
Waist circumference (cm)	94.7 ± 13.9	93.7 ± 13.8	0.43
Intracranial volume (cm^3)	1441.4 ± 142.7	1427.2 ± 139.7	0.41
**Blood pressure**			
Systolic blood pressure (mm Hg)	123.1 ± 14.5	120.0 ± 13.9	0.05
Diastolic blood pressure (mm Hg)	68.7 ± 8.8	75.2 ± 8.9	<0.001
Any hypertension (%)	86.5	22.2	<0.001
**Lipids (mg/dl)**			
Total cholesterol	170.8 ± 35.7	197.6 ± 40.8	<0.001
Triglycerides	70.0 ± 42.6	96.3 ± 47.7	<0.001
HDL cholesterol	65.5 ± 20.7	61.5 ± 19.1	0.08

© 2023 Habes M et al. *JAMA Network Open.*

LDL cholesterol	91.4 ± 29.4	117.0 ± 35.0	<0.001
Any hyperlipidemia (%)	86.0	24.2	<0.001
**Glycemia**			
HbA1c (%)	7.7 ± 1.1	5.5 ± 0.3	<0.001

[a] Data are mean ± SD or percent. Differences between the participants and controls were tested using the Wilcoxon rank-sum test for quantitative characteristics or chi-square test for categorical characteristics.

© 2023 Habes M et al. *JAMA Network Open.*

eTable 2. Differences in regions of interest (ROI) volumes between EDIC participants and controls without diabetes. Regions are listed in order of greatest effect size to least effect size.

Index	Region	Tissue	Effect Size [a]	FDR-Corrected-p-value
185	Left PT planum temporale	GM	5.35	0.000
184	Right PT planum temporale	GM	5.10	0.000
196	Right SOG superior occipital gyrus	GM	5.10	0.000
60	Left Thalamus Proper	GM	4.38	0.000
206	Right TTG transverse temporal gyrus	GM	4.34	0.000
197	Left SOG superior occipital gyrus	GM	4.26	0.000
58	Left Putamen	GM	4.25	0.000
59	Right Thalamus Proper	GM	4.22	0.000
56	Left Pallidum	GM	4.08	0.001
55	Right Pallidum	GM	4.06	0.001
30	Left Accumbens Area	GM	4.00	0.001
57	Right Putamen	GM	3.96	0.001
153	Left MSFG superior frontal gyrus medial segment	GM	3.91	0.001
23	Right Accumbens Area	GM	3.91	0.001
181	Left PP planum polare	GM	3.86	0.001
162	Right OpIFG opercular part of the inferior frontal gyrus	GM	3.80	0.001
91	anterior limb of internal capsule right	WM	3.78	0.001
137	Left LOrG lateral orbital gyrus	GM	3.73	0.001
205	Left TrIFG triangular part of the inferior frontal gyrus	GM	3.72	0.001
198	Right SPL superior parietal lobule	GM	3.68	0.002
113	Left CO central operculum	GM	3.59	0.002
92	anterior limb of internal capsule left	WM	3.58	0.002
174	Right PO parietal operculum	GM	3.54	0.002
175	Left PO parietal operculum	GM	3.46	0.003
200	Right STG superior temporal gyrus	GM	3.45	0.003
93	posterior limb of internal capsule inc. cerebral peduncle right	WM	3.43	0.003

© 2023 Habes M et al. *JAMA Network Open.*

190	Right SFG superior frontal gyrus	GM	3.40	0.003
106	Right AnG angular gyrus	GM	3.40	0.003
62	Left Ventral DC	WM	3.30	0.004
204	Right TrIFG triangular part of the inferior frontal gyrus	GM	3.29	0.004
94	posterior limb of internal capsule inc. cerebral peduncle left	WM	3.22	0.005
129	Left IOG inferior occipital gyrus	GM	3.20	0.006
199	Left SPL superior parietal lobule	GM	3.19	0.006
207	Left TTG transverse temporal gyrus	GM	3.16	0.006
143	Left MFG middle frontal gyrus	GM	3.15	0.006
108	Right Calc calcarine cortex	GM	3.15	0.006
112	Right CO central operculum	GM	3.12	0.006
173	Left PIns posterior insula	GM	3.11	0.007
183	Left PrG precentral gyrus	GM	3.09	0.007
180	Right PP planum polare	GM	3.09	0.007
201	Left STG superior temporal gyrus	GM	3.06	0.007
61	Right Ventral DC	WM	3.05	0.007
152	Right MSFG superior frontal gyrus medial segment	GM	3.03	0.008
145	Left MOG middle occipital gyrus	GM	2.97	0.009
102	Right AIns anterior insula	GM	2.95	0.009
90	fornix left	WM	2.94	0.01
118	Right FO frontal operculum	GM	2.88	0.01
121	Left FRP frontal pole	GM	2.87	0.01
107	Left AnG angular gyrus	GM	2.86	0.01
182	Right PrG precentral gyrus	GM	2.85	0.01
95	corpus callosum	WM	2.82	0.01
103	Left AIns anterior insula	GM	2.81	0.01
84	occipital lobe WM left	WM	2.81	0.01
141	Left MFC medial frontal cortex	GM	2.81	0.01
119	Left FO frontal operculum	GM	2.79	0.01
168	Right PCu precuneus	GM	2.77	0.01

© 2023 Habes M et al. *JAMA Network Open.*

191	Left SFG superior frontal gyrus	GM	2.74	0.01
35	Brain Stem	NONE	2.73	0.02
163	Left OpIFG opercular part of the inferior frontal gyrus	GM	2.72	0.02
114	Right Cun cuneus	GM	2.71	0.02
100	Right ACgG anterior cingulate gyrus	GM	2.66	0.02
177	Left PoG postcentral gyrus	GM	2.64	0.02
37	Left Caudate	GM	2.64	0.02
86	parietal lobe WM left	WM	2.63	0.02
120	Right FRP frontal pole	GM	2.63	0.02
83	occipital lobe WM right	WM	2.61	0.02
169	Left PCu precuneus	GM	2.59	0.02
167	Left PCgG posterior cingulate gyrus	GM	2.54	0.02
172	Right PIns posterior insula	GM	2.53	0.02
76	Right Basal Forebrain	GM	2.52	0.02
164	Right OrIFG orbital part of the inferior frontal gyrus	GM	2.44	0.03
85	parietal lobe WM right	WM	2.44	0.03
166	Right PCgG posterior cingulate gyrus	GM	2.42	0.03
75	Left Basal Forebrain	GM	2.40	0.03
109	Left Calc calcarine cortex	GM	2.39	0.03
192	Right SMC supplementary motor cortex	GM	2.35	0.03
88	temporal lobe WM left	WM	2.27	0.04
140	Right MFC medial frontal cortex	GM	2.26	0.04
41	Left Cerebellum White Matter	WM	2.23	0.04
176	Right PoG postcentral gyrus	GM	2.23	0.04
81	frontal lobe WM right	WM	2.16	0.05
36	Right Caudate	GM	2.15	0.05
157	Left OCP occipital pole	GM	2.12	0.06
40	Right Cerebellum White Matter	WM	2.11	0.06
193	Left SMC supplementary motor cortex	GM	2.11	0.06
87	temporal lobe WM right	WM	2.03	0.07

© 2023 Habes M et al. *JAMA Network Open.*

82	frontal lobe WM left	WM	2.03	0.07
155	Left MTG middle temporal gyrus	GM	2.02	0.07
195	Left SMG supramarginal gyrus	GM	1.97	0.08
154	Right MTG middle temporal gyrus	GM	1.95	0.08
104	Right AOrG anterior orbital gyrus	GM	1.92	0.08
101	Left ACgG anterior cingulate gyrus	GM	1.91	0.08
134	Right LiG lingual gyrus	GM	1.91	0.08
202	Right TMP temporal pole	GM	1.89	0.09
136	Right LOrG lateral orbital gyrus	GM	1.88	0.09
47	Right Hippocampus	GM	1.83	0.10
115	Left Cun cuneus	GM	1.81	0.10
31	Right Amygdala	GM	1.78	0.11
135	Left LiG lingual gyrus	GM	1.74	0.11
48	Left Hippocampus	GM	1.72	0.12
156	Right OCP occipital pole	GM	1.71	0.12
124	Right GRe gyrus rectus	GM	1.62	0.14
142	Right MFG middle frontal gyrus	GM	1.56	0.16
89	fornix right	WM	1.55	0.16
161	Left OFuG occipital fusiform gyrus	GM	1.53	0.17
194	Right SMG supramarginal gyrus	GM	1.50	0.17
32	Left Amygdala	GM	1.45	0.19
133	Left ITG inferior temporal gyrus	GM	1.35	0.23
138	Right MCgG middle cingulate gyrus	GM	1.34	0.23
144	Right MOG middle occipital gyrus	GM	1.32	0.24
125	Left GRe gyrus rectus	GM	1.21	0.28
105	Left AOrG anterior orbital gyrus	GM	1.17	0.30
128	Right IOG inferior occipital gyrus	GM	1.13	0.31
147	Left MOrG medial orbital gyrus	GM	1.12	0.32
203	Left TMP temporal pole	GM	1.05	0.35
139	Left MCgG middle cingulate gyrus	GM	0.90	0.44

© 2023 Habes M et al. *JAMA Network Open.*

39	Left Cerebellum Exterior	GM	0.75	0.54
73	Cerebellar Vermal Lobules VIII-X	GM	0.74	0.54
122	Right FuG fusiform gyrus	GM	0.73	0.54
187	Left SCA subcallosal area	GM	0.63	0.61
71	Cerebellar Vermal Lobules I-V	GM	0.63	0.61
132	Right ITG inferior temporal gyrus	GM	0.62	0.61
123	Left FuG fusiform gyrus	GM	0.57	0.63
38	Right Cerebellum Exterior	GM	0.56	0.64
186	Right SCA subcallosal area	GM	0.53	0.65
146	Right MOrG medial orbital gyrus	GM	0.51	0.66
165	Left OrIFG orbital part of the inferior frontal gyrus	GM	0.42	0.73
150	Right MPrG precentral gyrus medial segment	GM	0.39	0.75
148	Right MPoG postcentral gyrus medial segment	GM	0.34	0.78
151	Left MPrG precentral gyrus medial segment	GM	0.15	0.90
160	Right OFuG occipital fusiform gyrus	GM	0.13	0.91
171	Left PHG parahippocampal gyrus	GM	0.02	0.99
149	Left MPoG postcentral gyrus medial segment	GM	0.00	0.99
72	Cerebellar Vermal Lobules VI-VII	GM	-0.10	0.94
179	Left POrG posterior orbital gyrus	GM	-0.16	0.90
116	Right Ent entorhinal area	GM	-0.22	0.87
117	Left Ent entorhinal area	GM	-0.23	0.86
178	Right POrG posterior orbital gyrus	GM	-0.26	0.84
170	Right PHG parahippocampal gyrus	GM	-0.62	0.61
49	Right Inf Lat Vent	VN	-3.31	0.004
50	Left Inf Lat Vent	VN	-3.52	0.002
11	4th Ventricle	VN	-3.71	0.001
52	Left Lateral Ventricle	VN	-4.20	0.000
51	Right Lateral Ventricle	VN	-5.07	0.000
4	3rd Ventricle	VN	-6.99	0.000

[a] The signed effect sizes correspond to the magnitude and directionality of atrophy. Positive indicates more atrophy in EDIC participants.

© 2023 Habes M et al. *JAMA Network Open.*

eTable 3. Association of traditional glycemic and non-glycemic risk factors and micro- and macrovascular complications with MRI outcomes among EDIC participants, unadjusted (n=416)

	SPARE-AD [d]				SPARE-BA [d]			
	**β**	**SE**	**t**	**p-value**	**β**	**SE**	**t**	**p-value**
**Demographic Characteristics**								
Education (per 1 year)	0.00	0.03	-0.06	0.95	-0.47	0.24	-1.94	0.05
**Risk Factors**								
**Glycemic**								
HbA1c (per 1 %) [a]	-0.04	0.07	-0.55	0.58	-0.32	0.54	-0.59	0.55
Severe hypoglycemia								
Cumulative (≥1 vs. 0 events) [b]	-0.09	0.11	-0.85	0.40	0.38	0.91	0.41	0.68
1-5 vs. 0 events	-0.10	0.12	-0.80	0.42	0.80	0.97	0.82	0.41
>5 vs. 0 events	-0.09	0.20	-0.44	0.66	-1.28	1.62	-0.79	0.43
**Non-glycemic**								
Body mass index (per 1 kg/m^2) [a]	-0.04	0.02	-2.83	0.005	-0.29	0.12	-2.40	0.02
Waist circumference (per 5 cm)	-0.08	0.02	-3.88	<0.001	-0.31	0.16	-1.94	0.05
Blood pressure (per 5 mm Hg) [a]								
Systolic	-0.01	0.03	-0.22	0.83	1.14	0.27	4.21	<0.001
Diastolic	-0.09	0.05	-1.61	0.11	0.32	0.44	0.72	0.47
Any treated hypertension (yes vs. no)	-0.07	0.16	-0.44	0.66	3.92	1.30	3.01	0.003
Pulse rate (per 1 bpm) [a]	0.00	0.01	-0.58	0.56	-0.11	0.07	-1.58	0.12
Plasma lipids [a]								
HDL/LDL ratio (per 0.1)	0.06	0.03	2.26	0.02	0.21	0.22	0.94	0.35
Triglycerides (log)	-0.27	0.15	-1.83	0.07	-0.59	1.20	-0.49	0.62
Any treated hyperlipidemia (yes vs. no)	0.17	0.16	1.03	0.30	2.90	1.29	2.25	0.03
**Complications**								
**Kidney Disease**								
Sustained AER ≥30 mg/24 hr (yes vs. no) [c]	-0.19	0.13	-1.48	0.14	-1.46	1.06	-1.38	0.17
eGFR <60 mL/min/1.73 m^2 (yes vs. no) [c]	0.15	0.20	0.77	0.44	2.31	1.58	1.46	0.15
**Retinopathy**								

© 2023 Habes M et al. *JAMA Network Open.*

PDR (yes vs. no) [c]	-0.14	0.13	-1.09	0.28	-0.01	1.07	-0.01	0.99
CSME (yes vs. no) [c]	-0.19	0.12	-1.52	0.13	1.48	1.01	1.46	0.14
**Neuropathy**								
Confirmed clinical neuropathy (yes vs. no) [c]	-0.08	0.12	-0.66	0.51	3.14	0.99	3.16	0.002
Cardiovascular autonomic neuropathy (yes vs. no) [c]	0.23	0.11	1.99	0.05	3.63	0.90	4.02	<0.001
**Cardiovascular**								
Cardiovascular disease (yes vs. no) [c]	-0.03	0.17	-0.18	0.86	2.86	1.33	2.15	0.03

HDL denotes high-density lipoprotein, LDL low-density lipoprotein, AER albumin excretion rate, eGFR estimated glomerular filtration, PDR proliferative diabetic retinopathy, and CSME clinically-significant macular edema.

[a] Risk factors were characterized by the time-weighted mean values of all follow-up values since DCCT baseline up to the MRI study visit.

[b] Severe hypoglycemia was defined as events leading to coma or seizure documented by self-report for the 3-month period prior to each visit.

[c] Any report between DCCT baseline and the MRI study visit.

[d] Data are beta coefficients, standard errors, t-values, and p-values from individual linear regression models evaluating the association of each covariate of interest (independent) with each MRI outcome (dependent), unadjusted. Beta estimates are equal to the difference in means between groups or the slope of the association (e.g. increase or decrease in MRI outcome for every unit change in the covariate). The signed t-value corresponds to the magnitude and directionality of the association.

© 2023 Habes M et al. *JAMA Network Open.*

eTable 4. Association of HbA1c, SBP, and hypoglycemia with paired and unpaired ROI's (EDIC participants only, n=416)

		**HbA1c (per 1%)** [a]				**SBP (per 5 mm Hg)** [a]				**Cumulative Severe Hypo (≥1 vs. 0 events)** [b]			
		**β**	**SE**	**t**	**p**	**β**	**SE**	**t**	**p**	**β**	**SE**	**t**	**P**
**Paired** [c]	TTG transverse temporal gyrus	-0.015	0.015	-1.02	0.31	-0.013	0.008	-1.64	0.10	0.000	0.025	0.01	0.99
	TrIFG triangular part of the inferior frontal gyrus	-0.006	0.023	-0.25	0.81	-0.008	0.013	-0.64	0.52	-0.030	0.039	-0.76	0.45
	STG superior temporal gyrus	0.011	0.038	0.29	0.77	-0.013	0.021	-0.63	0.53	-0.010	0.064	-0.16	0.87
	SPL superior parietal lobule	-0.023	0.054	-0.42	0.67	-0.016	0.030	-0.55	0.58	-0.039	0.091	-0.43	0.67
	SOG superior occipital gyrus	-0.012	0.029	-0.42	0.68	-0.007	0.016	-0.45	0.65	-0.050	0.048	-1.03	0.30
	PT planum temporale	-0.018	0.015	-1.24	0.22	-0.011	0.008	-1.31	0.19	-0.022	0.025	-0.88	0.38
	PP planum polare	-0.003	0.011	-0.27	0.79	0.003	0.006	0.45	0.65	0.000	0.018	0.02	0.98
	PO parietal operculum	-0.020	0.017	-1.16	0.25	-0.012	0.009	-1.25	0.21	-0.004	0.029	-0.14	0.89
	MSFG superior frontal gyrus medial segment	-0.012	0.041	-0.30	0.77	-0.023	0.023	-1.02	0.31	0.040	0.070	0.58	0.56
	CO central operculum	-0.037	0.023	-1.59	0.11	-0.015	0.013	-1.15	0.25	0.030	0.039	0.77	0.44
	Thalamus Proper	-0.017	0.029	-0.58	0.56	0.004	0.016	0.26	0.79	-0.178	0.048	-3.74	<0.001
	Putamen	0.008	0.021	0.37	0.71	-0.008	0.012	-0.70	0.48	-0.009	0.036	-0.25	0.81
	Pallidum	0.002	0.007	0.26	0.79	0.002	0.004	0.47	0.64	-0.017	0.012	-1.35	0.18
	Accumbens Area	-0.002	0.003	-0.67	0.50	-0.002	0.002	-1.30	0.19	-0.001	0.005	-0.19	0.85
**Unpaired** [c]	Right SFG superior frontal gyrus	-0.145	0.072	-2.01	0.04	-0.060	0.040	-1.51	0.13	-0.097	0.122	-0.80	0.43
	Left PrG precentral gyrus	-0.042	0.067	-0.62	0.53	-0.012	0.037	-0.32	0.75	-0.321	0.113	-2.85	0.005
	Left PIns posterior insula	-0.029	0.014	-2.08	0.04	0.001	0.008	0.08	0.94	0.040	0.024	1.67	0.10
	Right OpIFG opercular part of the inferior frontal gyrus	-0.017	0.031	-0.57	0.57	0.003	0.017	0.20	0.84	0.074	0.052	1.44	0.15
	Left MFG middle frontal gyrus	-0.132	0.096	-1.37	0.17	-0.053	0.053	-0.99	0.32	-0.015	0.163	-0.09	0.93
	Left LOrG lateral orbital gyrus	0.015	0.022	0.66	0.51	-0.020	0.012	-1.63	0.10	-0.045	0.037	-1.20	0.23
	Left IOG inferior occipital gyrus	-0.027	0.043	-0.63	0.53	-0.011	0.023	-0.48	0.63	0.074	0.072	1.03	0.30
	Right Calc calcarine cortex	0.010	0.032	0.30	0.76	-0.030	0.017	-1.71	0.09	-0.054	0.053	-1.02	0.31
	Right AnG angular gyrus	-0.098	0.070	-1.40	0.16	-0.020	0.039	-0.51	0.61	-0.044	0.118	-0.37	0.71

[a] Risk factors were characterized by the time-weighted mean values of all follow-up values since DCCT baseline up to the MRI study visit.
[b] Severe hypoglycemia was defined as events leading to coma or seizure documented by self-report for the 3-month period prior to each visit.

© 2023 Habes M et al. *JAMA Network Open.*

[c] For the 14-paired regions, left and right regions were combined by taking the average. Data are beta coefficients, standard errors, t-values, and p-values from individual linear regression models evaluating the association of HbA1c (independent) with each ROI (dependent), with adjustment for ICV, age, sex, and scanner. Beta estimates are equal to the slope of the association (e.g. increase or decrease in ROI outcome for every unit change in the covariate). The signed t-value corresponds to the magnitude and directionality of the association.

© 2023 Habes M et al. *JAMA Network Open.*

eTable 5. Association of paired ROI's with Immediate Memory and Psychomotor and Mental Efficiency (EDIC participants only, n=415)

**Paired Region[a]**	**Immediate Memory**					**Psychomotor and Mental Efficiency**				
	**β**	**SE**	**t**	**p-value**	**FDR-Corrected-p-value**	**B**	**SE**	**t**	**p-value**	**FDR-Corrected-p-value**
TTG transverse temporal gyrus	0.295	0.174	1.70	0.09	0.21	0.398	0.207	1.92	0.05	0.08
TrIFG triangular part of the inferior frontal gyrus	0.103	0.110	0.94	0.35	0.54	0.257	0.131	1.97	0.05	0.08
STG superior temporal gyrus	0.150	0.067	2.25	0.03	0.16	0.190	0.080	2.39	0.02	0.05
SPL superior parietal lobule	0.035	0.047	0.74	0.46	0.60	-0.022	0.057	-0.39	0.70	0.70
SOG superior occipital gyrus	0.073	0.090	0.82	0.41	0.59	0.093	0.107	0.87	0.38	0.40
PT planum temporale	0.414	0.173	2.39	0.02	0.16	0.732	0.205	3.57	<0.001	0.00
PP planum polare	-0.135	0.238	-0.57	0.57	0.63	0.462	0.283	1.64	0.10	0.11
PO parietal operculum	0.433	0.149	2.91	0.004	0.09	0.682	0.176	3.88	<0.001	0.001
MSFG superior frontal gyrus medial segment	0.011	0.062	0.18	0.86	0.86	0.138	0.074	1.88	0.06	0.08
CO central operculum	0.074	0.110	0.67	0.50	0.61	0.272	0.130	2.08	0.04	0.06
Thalamus Proper	0.183	0.089	2.05	0.04	0.16	0.419	0.105	4.01	<0.001	0.001
Putamen	0.112	0.119	0.94	0.35	0.54	0.249	0.142	1.76	0.08	0.10
Pallidum	-0.254	0.351	-0.72	0.47	0.60	0.883	0.417	2.12	0.03	0.06
Accumbens Area	1.274	0.891	1.43	0.15	0.32	3.465	1.052	3.29	0.001	0.005
**Unpaired Region [a]**										
Right SFG superior frontal gyrus	0.075	0.035	2.13	0.03	0.16	0.123	0.042	2.93	0.004	0.01
Left PrG precentral gyrus	0.074	0.038	1.96	0.05	0.16	0.079	0.045	1.74	0.08	0.10
Left PIns posterior insula	0.345	0.181	1.91	0.06	0.16	0.498	0.215	2.32	0.02	0.05
Right OpIFG opercular part of the inferior frontal gyrus	0.048	0.083	0.58	0.56	0.63	0.204	0.099	2.07	0.04	0.06
Left MFG middle frontal gyrus	0.050	0.026	1.88	0.06	0.16	0.108	0.031	3.48	<0.001	0.003
Left LOrG lateral orbital gyrus	0.144	0.116	1.24	0.22	0.41	0.288	0.138	2.09	0.04	0.06
Left IOG inferior occipital gyrus	0.117	0.060	1.95	0.05	0.16	0.164	0.072	2.30	0.02	0.05

© 2023 Habes M et al. *JAMA Network Open.*

Right Calc calcarine cortex	0.036	0.081	0.44	0.66	0.69	0.159	0.097	1.65	0.10	0.11
Right AnG angular gyrus	0.039	0.037	1.07	0.28	0.50	0.119	0.043	2.75	0.006	0.02

[a] For the 14-paired regions, left and right regions were combined by taking the average. Data are beta coefficients, standard errors, t-values, and p-values from individual linear regression models evaluating the association of each ROI (independent) with each cognitive domain (dependent), with adjustment for ICV, age, sex, years of education, and scanner. Beta estimates are equal to the slope of the association (e.g. increase or decrease in cognitive domain for every unit change in the covariate). The signed t-value corresponds to the magnitude and directionality of the association.

© 2023 Habes M et al. *JAMA Network Open.*

<u>DCCT/EDIC Research Group as of July 1, 2022</u>

Study Chairpersons – D.M. Nathan (chair), B. Zinman (vice-chair); *Past*: O. Crofford; *Deceased*: S. Genuth

Editor, EDIC Publications – D.M. Nathan

Clinical Centers

<u>Case Western Reserve University</u> – *Current*: R. Gubitosi-Klug, L. Mayer, J. Wood, G. Greanoff, D. Miller, M. Novak, S. Pendegast, S. Rath, L. Singerman, D. Weiss, H. Zegarra; *Past*: E. Brown, P. Crawford, M. Palmert, P. Pugsley, J. Quin, S. Smith-Brewer; *Deceased*: W. Dahms, S. Genuth, J. McConnell

<u>Weill Cornell Medical College</u> – *Current*: N.S. Gregory, R. Hanna, R. Chan, S. Kiss, A. Orlin, M. Rubin; *Past*: S. Barron, B. Bosco, D. Brillon, S. Chang, A. Dwoskin, M. Heinemann, L. Jovanovic, M.E. Lackaye, T. Lee, B. Levy, V. Reppucci, M. Richardson; *Deceased*: R. Campbell

<u>Henry Ford Health System</u> – *Current*: A. Bhan, J.K. Jones, D. Kruger, P.A. Edwards, S. Mukhashen; *Past*: E. Angus, A. Galprin, M. McLellan, H. Remtema, A. Thomas; *Deceased*: J.D. Carey, F. Whitehouse

<u>International Diabetes Center</u> – *Current*: R. Bergenstal, S. Dunnigan, M. Johnson, A. Carlson, L. Thomas; *Past*: R. Birk, P. Callahan, G. Castle, R. Cuddihy, M. Franz, D. Freking, L. Gill, J. Gott, K. Gunyou, P. Hollander, D. Kendall, J. Laechelt, S. List, G. Matfin, W. Mestrezat, J. Nelson, B. Olson, N. Rude, M. Spencer; *Deceased*: D. Etzwiler, K. Morgan

<u>Joslin Diabetes Center</u> – *Current*: L.P. Aiello, E. Golden, P. Arrigg, R. Beaser, J. Cavallerano, R. Cavicchi, O. Ganda, O. Hamdy, T. Murtha, D. Schlossman, S. Shah, G. Sharuk, P. Silva, P. Silver, M. Stockman, J. Sun, E. Weimann; *Past*: V. Asuquo, L. Bestourous, A. Jacobson, R. Kirby, L. Rand, J. Rosenzwieg, H. Wolpert

<u>Massachusetts General Hospital</u> – *Current*: D.M. Nathan, M.E. Larkin, K. Chu, J. Heier, A. Joseph, F. Leandre, C. Shah, N. Thangthaeng; *Past*: E. Anderson, H. Bode, S. Brink, M. Cayford, M. Christofi, C. Cornish, D. Cros, S. Crowell, L. Delahanty, A. deManbey, K. Folino, S. Fritz, C. Gauthier-Kelly, J. Godine, L. Gurry, C. Haggan, K. Hansen, P. Lou, J. Lynch, K. Martin, C. McKitrick, D. Moore, D. Norman, M. Ong, E. Ryan, C. Stevens, C. Taylor, D. Zimbler

<u>Mayo Clinic</u> – *Current*: A. Vella, A. Zipse, A. Barkmeier; *Past*: B. French, M. Haymond, J. Mortenson, J. Pach, R. Rizza, L. Schmidt, W.F. Schwenk, R. Woodwick, G. Ziegler; *Deceased*: R. Colligan, A. Lucas, F.J. Service, B. Zimmerman

<u>Medical University of South Carolina</u> – *Current*: H. Karanchi, L. Spillers, J. Fernandes, K. Hermayer, K. Lee, M. Lopes-Virella, T. Lyons, M. Nutaitis; *Past*: A. Blevins, M. Bracey, S. Caulder, J. Colwell, S. Elsing, A. Farr, S. Kwon, D. Lee, P. Lindsey, L. Luttrell, R. Mayfield,

© 2023 Habes M et al. *JAMA Network Open.*

J. Parker, N. Patel, C. Pittman, J. Selby, J. Soule, M. Szpiech, T. Thompson, D. Wood, S. Yacoub-Wasef

Northwestern University – *Current*: A. Wallia, M. Hartmuller, S. Ajroud-Driss, P. Astelford, A. Degillio, M. Gill, L. Jampol, C. Johnson, L. Kaminski, N. Leloudes, A. Lyon, R. Mirza, D. Ryan, E. Simjanoski, Z. Strugula; *Past*: D. Adelman, S. Colson, M. Molitch, B. Schaefer

University of California, San Diego – *Current*: S. Mudaliar, G. Lorenzi, O. Kolterman, M. Goldbaum; *Past*: T. Clark, M. Giotta, I. Grant, K. Jones, R. Lyon, M. Prince, R. Reed, M. Swenson; *Deceased*: G. Friedenberg

University of Iowa – *Current*: W.I. Sivitz, B. Vittetoe; *Past*: M. Bayless, C. Fountain, R. Hoffman, J. Kramer, J. MacIndoe, N. Olson, H. Schrott, L. Snetselaar, T. Weingeist, R. Zeitler

University of Maryland – *Current*: R. Miller, S. Johnsonbaugh; *Past*: M. Carney, D. Counts, T. Donner, J. Gordon, M. Hebdon, R. Hemady, B. Jones, A. Kowarski, R. Liss, S. Mendley, D. Ostrowski, M. Patronas, P. Salemi, S. Steidl

University of Michigan – *Current*: W.H. Herman, R. Pop-Busui, C.L. Martin, P. Lee, J. W. Albers, E.L. Feldman; *Past*: N. Burkhart, D.A. Greene, T. Sandford, M.J. Stevens; *Deceased*: J. Floyd

University of Minnesota – *Current*: A. Bantle, J. Bantle, M. Rhodes, D. Koozekanani, S. Montezuma, J. Terry; *Past*: N. Flaherty, F. Goetz, C. Kwong, L. McKenzie, M. Mech, J. Olson, B. Rogness, T. Strand, J. Terry, R. Warhol, N. Wimmergren

University of Missouri – *Current*: D. Goldstein, D. Hainsworth, S. Hitt, A. Jarvis; *Deceased*: J. Giangiacomo

University of New Mexico – *Current*: D.S. Schade, A. Bancroft, R.B. Avery, M.R. Burge, J.E. Chapin, A. Das, L.H. Ketai; *Past*: J.L. Canady, D. Hornbeck, C. Johannes, J. Rich, M.L Schluter

University of Pennsylvania – *Current*: M. Schutta, P.A. Bourne, A. Brucker; *Past*: S. Braunstein, B.J. Maschak-Carey, S. Schwartz; *Deceased*: L. Baker

University of Pittsburgh – *Current*: T. Costacou, F. Toledo, T. Orchard, B.A. Coonrod; *Past*: D. Becker, L. Cimino, B. Doft, D. Finegold, K. Kelly, L. Lobes, D. Rubinstein, N. Silvers, T. Songer, D. Steinberg, L. Steranchak, J.Wesche; *Deceased*: A. Drash

University of South Florida – *Current*: J.I. Malone, A. Morrison, H. Rodriguez, J. O'Brian, P.R. Pavan; *Past*: L. Babbione, M.L. Bernal,T.J. DeClue, N. Grove, D. McMillan, H. Solc, E.A. Tanaka, J. Vaccaro-Kish

University of Tennessee – *Current*: S. Dagogo-Jack, R. Wilson, S. Huddleston; *Past*: M. Bryer-Ash, E. Chaum, A. Iannacone, H. Lambeth, D. Meyer, S. Moser, M.B. Murphy, A. Patel, H. Ricks, S. Schussler, C. Wigley, S. Yoser; *Deceased*: A. Kitabchi

© 2023 Habes M et al. *JAMA Network Open.*

University of Texas – *Current*: P. Raskin, L. Jordan, YG. He, E. Mendelson, RL. Ufret-Vincenty; *Past*: M. Basco; *Deceased*: S. Cercone, S. Strowig

University of Toronto – *Current*: B.A. Perkins, A. Barnie, N. Bakshi, M. Brent, R. Devenyi, K. Koushan, M. Mandelcorn, D. Olegario, F. Perdikaris; *Past*: D. Daneman, R. Ehrlich, S. Ferguson, A. Gordon, K. Perlman, S. Rogers, L. Tuason, B. Zinman

University of Washington – *Current*: I. Hirsch, R. Fahlstrom, L. Van Ottingham, I.H. de Boer, L. Olmos de Koo; *Past*: S. Catton, J. Ginsberg, J. Kinyoun, J. Palmer

University of Western Ontario – *Current*: C. McDonald, M. Driscoll, J. Bylsma, T. Sheidow; *Past*: W. Brown, C. Canny, P. Colby, S. Debrabandere, J. Dupre, J. Harth, I. Hramiak, M. Jenner, J. Mahon, D. Nicolle, N.W. Rodger, T. Smith

Vanderbilt University – *Current*: M. May, T. Marksbury, T. Adkins, A. Agarwal, C. Lovell; *Past*: S. Feman, J. Lipps Hagan, R. Lorenz, R. Ramker; *Deceased*: L. Survant

Washington University, St. Louis – *Current*: N.H. White, E. Hoffman; *Past*: L. Levandoski; *Deceased*: I. Boniuk, J. Santiago

Yale University – *Current*: W. Tamborlane, P. Gatcomb, K. Stoessel; *Past*: J. Ahern

Albert Einstein – *Past*: J. Brown-Friday, J. Crandall, H. Engel, S. Engel, H. Martinez, M. Phillips, M. Reid, H. Shamoon, J. Sheindlin

Clinical Coordinating Center

Case Western Reserve University – *Current*: R. Gubitosi-Klug, L. Mayer, K. Farrell; *Past*: C. Beck, P. Gaston, M. Palmert, J. Quin, R. Trail; *Deceased*: W. Dahms, S. Genuth

Data Coordinating Center

George Washington University, The Biostatistics Center – *Current*: J. Lachin, I. Bebu, B. Braffett, J. Backlund, M. Bott, L. Diminick, L. El ghormli, X. Gao, S. Ho, D. Kenny, K. Klumpp, M. Lin, V. Trapani; *Past*: K. Anderson, K. Chan, P. Cleary, A. Determan, L. Dews, W. Hsu, P. McGee, H. Pan, B. Petty, D. Rosenberg, B. Rutledge, W. Sun, S. Villavicencio, N. Younes; *Deceased*: C. Williams

National Institute of Diabetes and Digestive and Kidney Disease

National Institute of Diabetes and Digestive and Kidney Disease Program Office – *Current*: E. Leschek; *Past*: C. Cowie, C. Siebert

EDIC Core Central Units

Central Biochemistry Laboratory (University of Minnesota) – *Current*: M. Steffes, A. Karger, J. Seegmiller, V. Arends; *Past*: J. Bucksa, B. Chavers, A. Killeen, M. Nowicki, A. Saenger

© 2023 Habes M et al. *JAMA Network Open.*

Central ECG Reading Unit (Wake Forest School of Medicine) – *Current*: E.Z. Soliman, M. Barr, C. Campbell, S. Hensley, J. Hu, L. Keasler, Y. Li, T. Taylor, Z.M. Zhang; *Past*: Y. Pokharel, R. Prineas

Central Ophthalmologic Reading Unit (University of Wisconsin) – *Current*: B. Blodi, R. Danis, D. Lawrence, H. Wabers; *Past*: M. Burger, M. Davis, J. Dingledine, V. Gama, S. Gangaputra, L. Hubbard, S. Neill, R. Sussman

Central Neuropsychological Reading Unit (NYU Long Island School of Medicine, University of Pittsburgh) – *Current*: A. Jacobson, C. Ryan, D. Saporito; *Past*: B. Burzuk, E. Cupelli, M. Geckle, D. Sandstrom, F. Thoma, T. Williams, T. Woodfill

© 2023 Habes M et al. *JAMA Network Open.*
