# Supplementary material for: Patterns of Regional Brain Atrophy and Brain Aging in Middle- and Older-Aged Adults With Type 1 Diabetes
Source: JAMA Netw Open. 2023 Jun 1;6(6):e2316182. doi: 10.1001/jamanetworkopen.2023.16182 (PMC10236234; doi:10.1001/jamanetworkopen.2023.16182)
Supplement: Supplement 2. — Data Sharing Statement [file jamanetwopen-e2316182-s002.pdf]

Data Sharing Statement

Habes. Patterns of Regional Brain Atrophy and Brain Aging in Middle- and Older-Aged Adults With Type 1 Diabetes. *JAMA Netw Open*. Published June 01, 2023. doi:10.1001/jamanetworkopen.2023.16182

Data
Data available: Yes
Data types: Deidentified participant data
How to access data: NIDDK Central Repository
When available: With publication

Supporting Documents
Document types: None

Additional Information
Who can access the data: Anyone requesting data that has approval from the NIDDK Central Repository
Types of analyses: For any purpose approved by the NIDDK Central Repository
Mechanisms of data availability: After approval by the NIDDK Central Repository
